# Supplementary material for: Evaluating methods and protocols of ferritin-based magnetogenetics
Source: iScience. 2021 Sep 9;24(10):103094. doi: 10.1016/j.isci.2021.103094 (PMC8479696; doi:10.1016/j.isci.2021.103094)
Supplement: Document S1. Figures S1–S4 and Table S1 [file mmc1.pdf]

iScience, Volume *24*

Supplemental information

Evaluating methods and protocols of ferritin-based magnetogenetics

Miriam Hernández-Morales, Victor Han, Richard H. Kramer, and Chunlei Liu

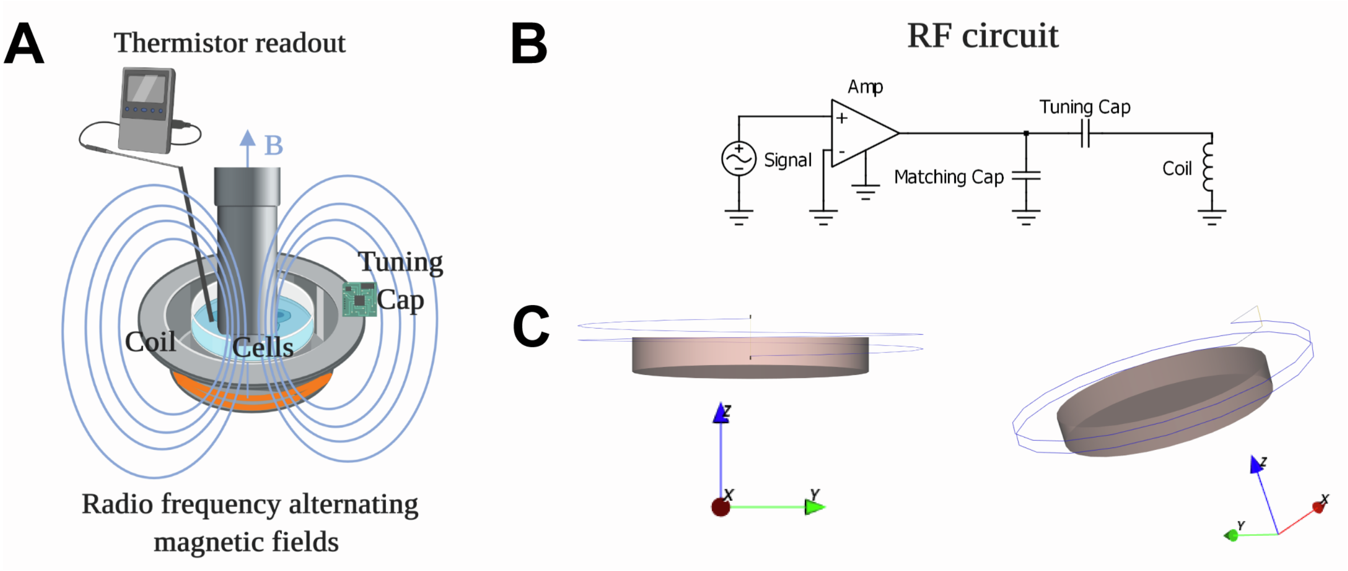

Figure S1. Scheme of the RF setup. Related to Figure 1. (**A**) Illustration of the experimental set up for RF stimulation of cultured cells. (**B**) Simplified RF circuit. (**C**) Two orientations of 3D models of the culture dish and the solenoid RF coil.

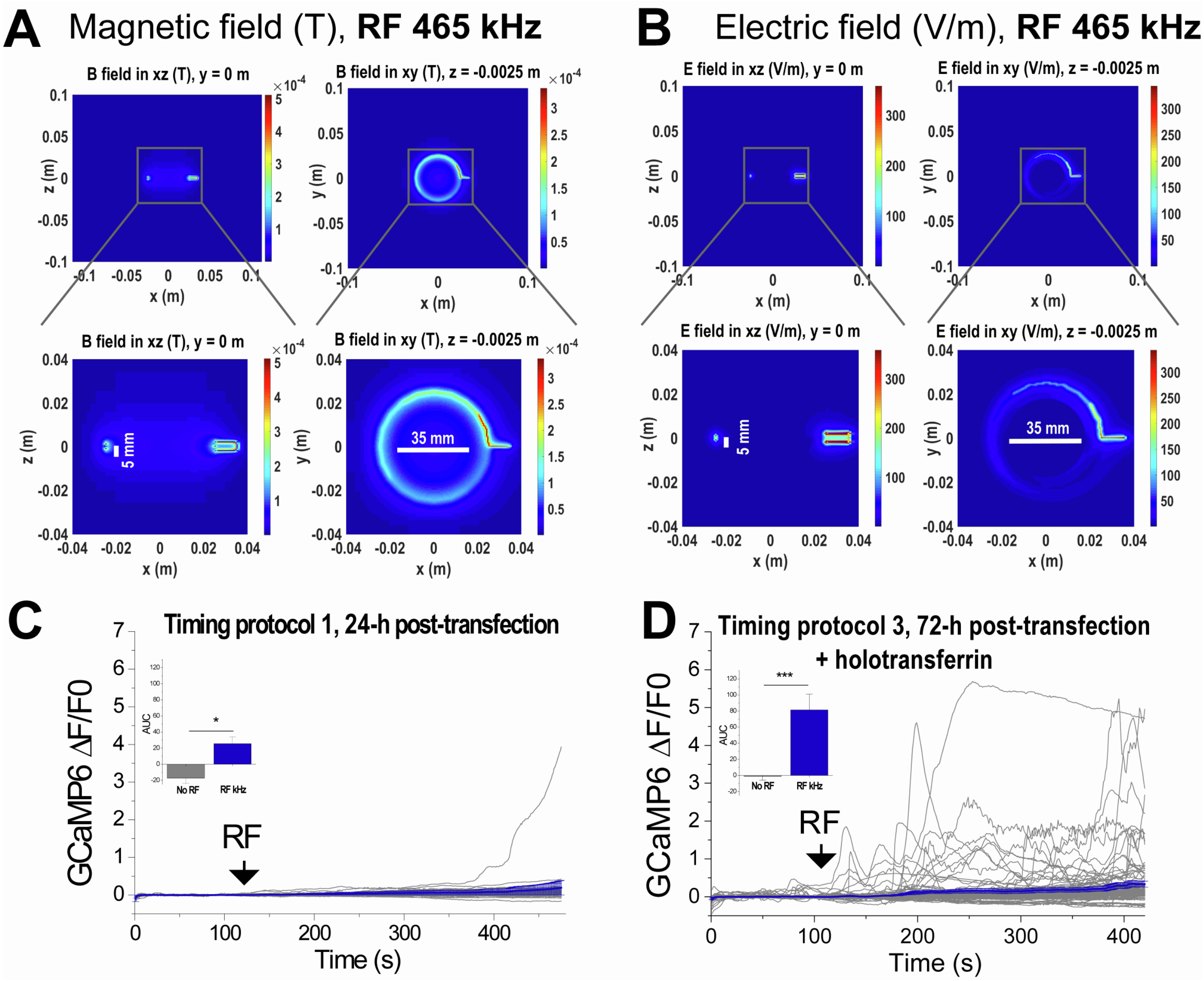

Figure S2. RF stimulation at kHz frequency at the µT range has limited efficiency for activating TRPV4$^{\mathbf{FeRIC}}$**. Related to Figures 1 and 2.** (**A**, **B**) Simulations of the magnetic and electric fields produced by RF at kHz. Distribution of the (**A**) magnetic and (**B**) electric fields in the culture dish produced by RF fields at 465 kHz and 31 µT; bottom: zoom-in of the culture dish. Vertical lines correspond to the 5mm-height of the saline solution being in the lower half of the coil. Horizontal lines: 35 mm-diameter of the culture dish. Cultured cells are at the center of the culture dish. (**C, D**) All changes and average changes (± SEM) in GCaMP6 ΔF/F0 in N2a cells expressing GCaMP6 plus TRPV4^{FeRIC} following stimulation with RF at 465 kHz and 31 µT for 6 min (black arrows). Cells were imaged (**C**) 24-h or (**D**) 72-h post-transfection. Cells in (D) were supplemented with 500 µM HTF after transfection. Insets: average changes (± SEM) in GCaMP6 AUC for the period of RF stimulation. Where applicable, either $p<0.05$ (*), $p<0.001$ (**), or $p<0.0001$ (***) was considered a statistically significant difference.

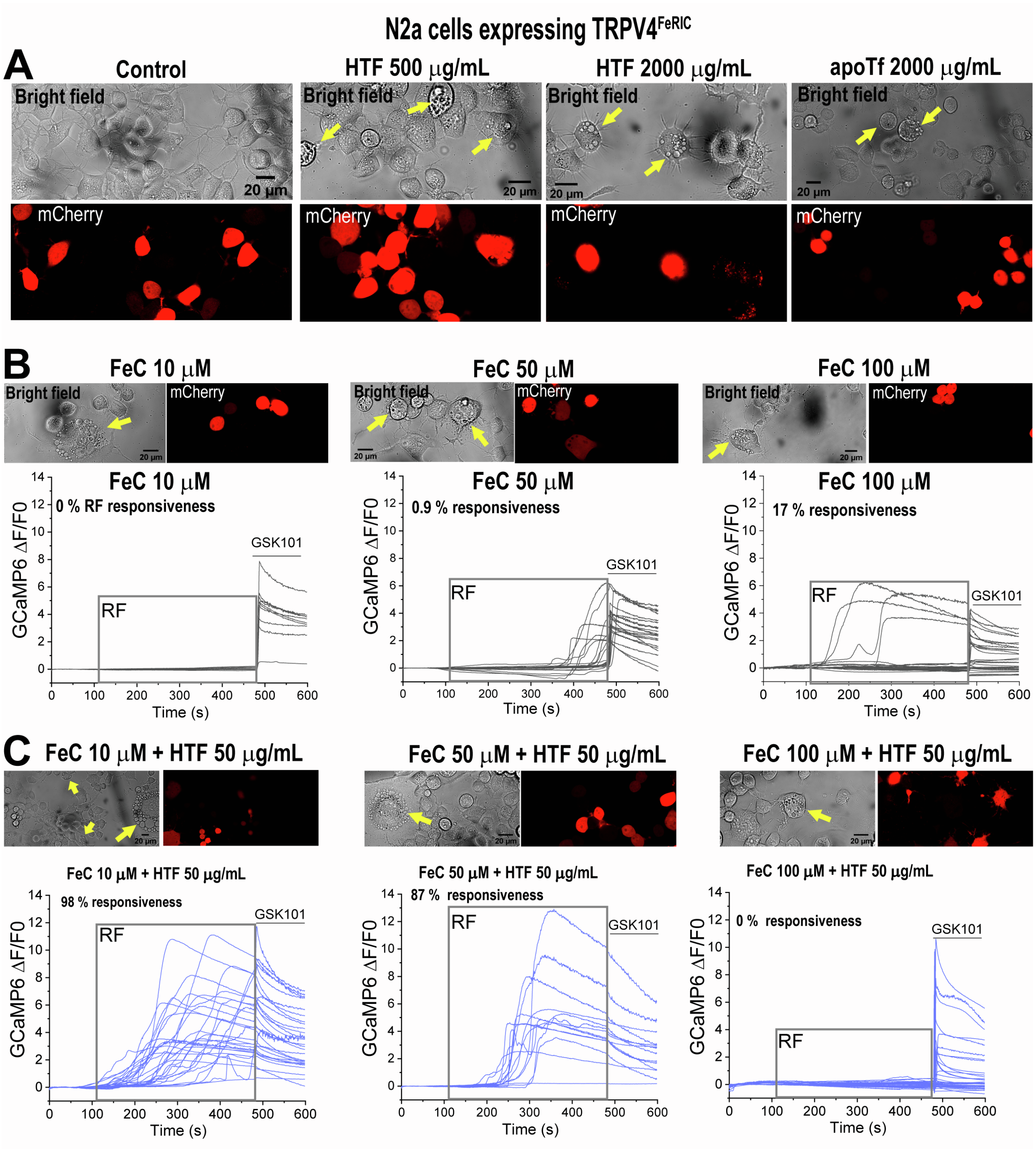

Figure S3. Increasing cellular iron import can increase the RF-induced activation of TRPV4^{FeRIC} but can also negatively affect cell health. Related to Figure 2. (**A**) Bright-field and fluorescence images of N2a cells expressing TRPV4^{FeRIC} (mCherry+). In separate experiments, cells were treated with holotransferrin (HTF, 500 or 2000 μg/mL) or apotransferrin (apoTf, 2000 μg/mL) after transfection. Examples of vacuolated cells are indicated with yellow arrows. (**B, C**) Top images are bright-field and fluorescence images of N2a cells expressing TRPV4^{FeRIC} (mCherry+). Bottom plots are changes in GCaMP6 ΔF/F0 in N2a cells expressing TRPV4^{FeRIC} upon RF stimulation (gray box, 180 MHz, 1.6 μT), followed by GSK101 (solid line). In separate experiments cells were treated with (**B**) ferric citrate (FeC at 10, 50, and 100 μM) or (**C**) a combination of HTF (50 μg/mL) and FeC (10, 50, and 100 μM) after transfection. Examples of vacuolated cells are indicated with yellow arrows. Scale bars = 20 μm.

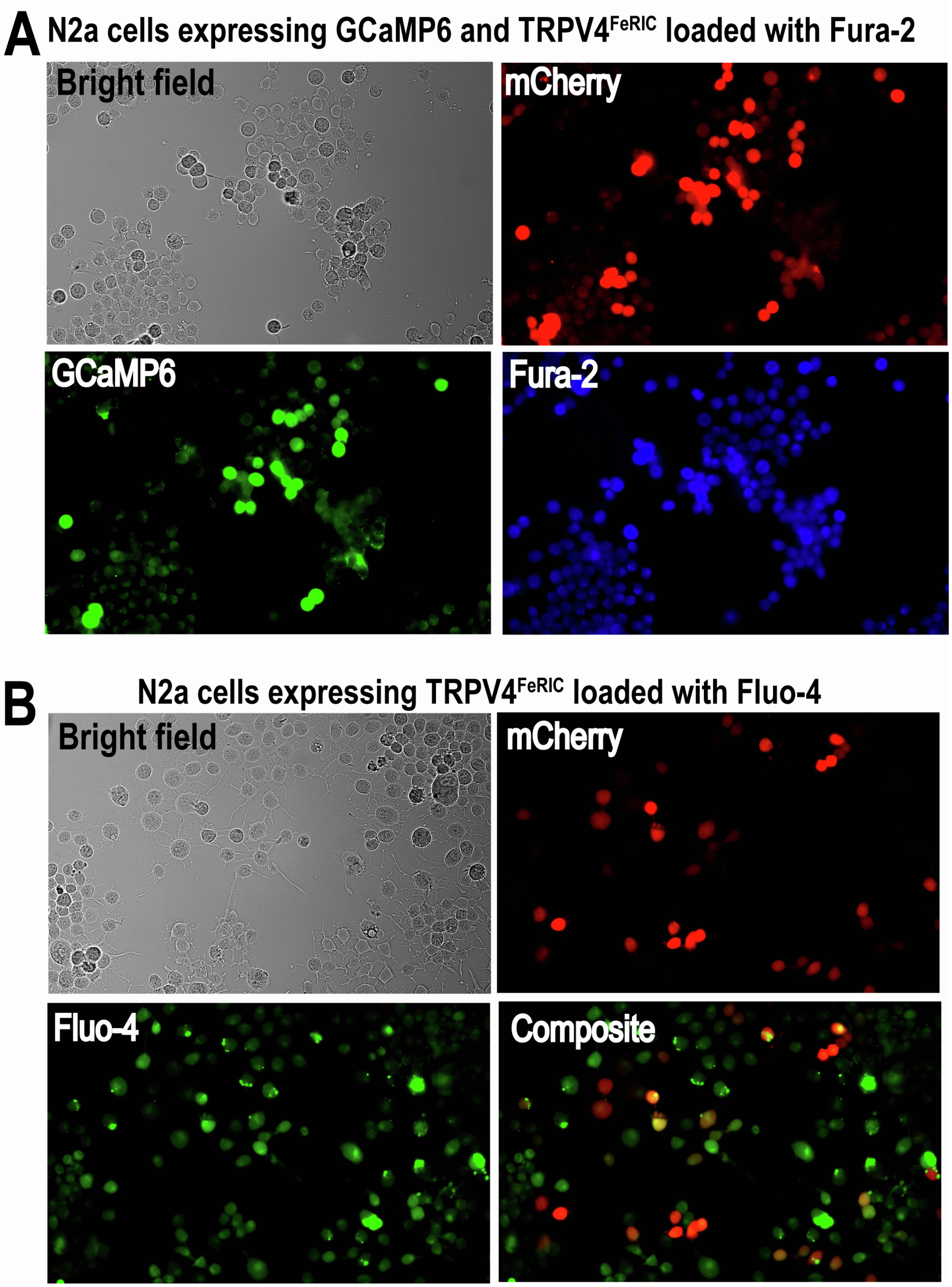

Figure S4. RF-induced activation of TRPV4^{FeRIC} in N2a cells expressing TRPV4^{FeRIC} loaded with Ca^{2+} dyes. Related to Figure 4. (**A**) Representative images of N2a cells expressing GCaMP6 plus TRPV4^{FeRIC} (mCherry^{+}) and loaded with Fura-2 (1 μM). (**B**) Representative images of N2a cells expressing TRPV4^{FeRIC} (mCherry^{+}) and loaded with Fluo-4 (1 μM).

Experimental group	AUC (± SEM)	Fraction of responsive cells (± SEM)	N= number independent experiments	n= number of cells
**Neuro2a cells expressing GCaMP6 plus TRPV4^{FeRIC} – Timing protocol 1 – 24 h post-transfection**				
TRPV4^{FeRIC} - No RF	-17.4 ± 6.1	0.19 ± 0.11	4	161
TRPV4^{FeRIC}- RF 1.6 μT (180 MHz)	226.4 ± 31.4	0.43 ± 0.12	5	289
TRPV4^{FeRIC}- RF 1.6 μT (180 MHz) + GSK219	16.8 ± 9.9	0.19 ± 0.04	4	94
TRPV4^{FeRIC}- RF 1.6 μT (180 MHz) Consecutive 1st stimulation	116.3 ± 23	0.26 ± 0.08	6	151
TRPV4^{FeRIC}- RF 1.6 μT (180 MHz) Consecutive 2nd stimulation	80.5 ± 23.5	0.33 ± 0.1	6	209
TRPV4^{FeRIC}- RF 1.6 μT (180 MHz) 1st stimulation at 22 ºC	263 ± 40.8	0.66 ± 12.6	4	56
TRPV4^{FeRIC}- RF 1.6 μT (180 MHz) 2nd stimulation at 32 ºC	122 ± 16.6	0.48 ± 9.7	4	56
TRPV4^{FeRIC}- RF 1.6 μT (180 MHz) 3rd stimulation at 37 ºC	126.2 ± 28.8	0.34 ± 9.2	4	56
**Neuro2a cells expressing GCaMP6 plus TRPV4^{FeRIC} – Timing protocol 1 – 24 h post-transfection plus holotransferrin (HTF)**				
TRPV4^{FeRIC} - No RF	31 ± 9.5	0.27 ± 0.06	5	113
TRPV4^{FeRIC}- RF 1.6 μT (180 MHz) + HTF 500 μg/mL	520 ± 50.2	0.52 ± 0.14	7	164
TRPV4^{FeRIC}- RF 1.6 μT (180 MHz) + 500 μg/mL + GSK219	10.2 ± 9.5	0.35 ± 0.18	5	38
TRPV4^{FeRIC}- RF 1.6 μT (180 MHz) + HTF 25 μg/Ml	128 ± 29.2	0.31 ± 0.27	3	104
TRPV4^{FeRIC}- RF 1.6 μT (180 MHz) + HTF 100 μg/mL	552.4 ± 40.1	0.48 ± 0.19	5	164
TRPV4^{FeRIC}- RF 1.6 μT (180 MHz) + HTF 2000 μg/mL	20.3 ± 7.5	0.14 ± 0.13	3	81
**Neuro2a cells expressing GCaMP6 plus TRPV4^{FeRIC} – Timing protocol 1 – 24 h post-transfection plus apotransferrin (apoTf)**				
TRPV4^{FeRIC}- RF 1.6 μT (180 MHz) + apoTf 100 μg/mL	106.9 ± 38.6	0.58 ± 0.04	3	74

TRPV4^{FeRIC}- RF 1.6 μT (180 MHz) + apoTf 500 μg/mL	643.5 ± 63.3	0.65 ± 0.2	3	133
TRPV4^{FeRIC}- RF 1.6 μT (180 MHz) + apoTf 2000 μg/mL	261.8 ± 35	0.5 ± 0.3	3	75
**Neuro2a cells expressing GCaMP6 plus TRPV4^{FeRIC} – Timing protocol 2 – 48 h post-**				
TRPV4^{FeRIC} - No RF	8.5 ± 12.9	0.23 ± 0.08	5	45
TRPV4^{FeRIC}- RF 1.6 μT (180 MHz)	95.5 ± 34	0.27 ± 0.10	6	67
TRPV4^{FeRIC}- RF 1.6 μT + (180 MHz) GSK219	40.4 ± 19	0.35 ± 0.17	6	55
**Neuro2a cells expressing GCaMP6 plus TRPV4^{FeRIC} – Timing protocol 2 – 48 h post-transfection plus HTF 500 μg/mL**				
TRPV4^{FeRIC} - No RF	-0.5 ± 7.3	0.14 ± 0.05	5	81
TRPV4^{FeRIC}- RF 1.6 μT	260.6 ± 52.9	0.43 ± 0.16	5	113
TRPV4^{FeRIC}- RF 1.6 μT + GSK219	-12.6 ± 6.8	0.07 ± 0.04	5	83
**Neuro2a cells expressing GCaMP6 plus TRPV4^{FeRIC} – Timing protocol 3 – 72 h post-**				
TRPV4^{FeRIC} - No RF	21.1 ± 5.8	0.12 ± 0.08	5	111
TRPV4^{FeRIC}- RF 1.6 μT (180 MHz)	48.1 ± 26.2	0.3 ± 0.07	9	114
TRPV4^{FeRIC}- RF 1.6 μT (180 MHz) + GSK219	-11.2 ± 6.3	0.02 ± 0.02	8	80
**Neuro2a cells expressing GCaMP6 plus TRPV4^{FeRIC} – Timing protocol 3 – 72 h post-transfection plus HTF 500 μg/mL**				
TRPV4^{FeRIC} - No RF	-1.2 ± 4.8	0.1 ± 0.03	11	329
TRPV4^{FeRIC}- RF 1.6 μT (180 MHz)	550.1 ± 51	0.66 ± 0.09	9	219
TRPV4^{FeRIC}- RF 1.6 μT (180 MHz) + GSK219	7.2 ± 15.8	0.1 ± 0.05	8	80
**Neuro2a cells expressing GCaMP6 plus TRPV4$^{\Delta TFeRIC}$ – Timing protocol 1 – 24 h post-**				
TRPV4$^{\Delta TFeRIC}$ - No RF	4.3 ± 2.2	0.23 ± 0.04	5	318
TRPV4$^{\Delta TFeRIC}$ - RF 1.6 μT (180	393.8 ± 62.5	0.52 ± 0.06	4	236
TRPV4$^{\Delta TFeRIC}$ - RF 1.6 μT + (180 MHz) GSK219	-15.8 ± 6.9	0.1 ± 0.03	4	167
**Neuro2a cells expressing GCaMP6 plus TRPV4$^{\Delta TFeRIC}$ – Timing protocol 3 – 72 h post-**				
TRPV4$^{\Delta TFeRIC}$ - No RF	7.8 ± 3.4	0.27 ± 0.08	4	70
TRPV4$^{\Delta TFeRIC}$ - RF 1.6 μT (180	399.6 ± 42.4	0.57 ± 0.13	6	247
TRPV4$^{\Delta TFeRIC}$ - RF 1.6 μT (180 MHz) + GSK219	-17.2 ± 8.8	0.37 ± 0.23	4	53
**Neuro2a cells expressing GCaMP6 plus TRPV4^{FeRIC} – Timing protocol 1 – 24 h post-transfection –EGTA treatment**				
TRPV4^{FeRIC} - No RF + EGTA	-5.5 ± 13	0.28 ± 0.14	3	29
TRPV4^{FeRIC}- RF 1.6 μT (180 MHz) + EGTA	526.2 ± 90.4	0.53 ± 0.16	4	93

**Neuro2a cells expressing GCaMP6 plus TRPV4^{FeRIC} – Timing protocol 3 – 72 h post-transfection –EGTA treatment**				
TRPV4^{FeRIC} - No RF + EGTA	1.46 ± 5.4	0.1 ± 0.07	5	89
TRPV4^{FeRIC}- RF 1.6 μT (180 MHz) + EGTA	209.9 ± 52.8	0.32 ± 0.08	7	102
**Neuro2a cells expressing GCaMP6 plus TRPV4^{FeRIC} – Timing protocol 1 – 24 h post-transfection + Fura-2**				
TRPV4^{FeRIC} - RF 1.6 μT (180 MHz)	-4.3 ± 3.9	0.17 ± 0.04	4	202
**Neuro2a cells expressing TRPV4^{FeRIC} loaded with Fluo-4 – Timing protocol 1 – 24 h post-transfection**				
TRPV4^{FeRIC} - RF 1.6 μT (180 MHz)	215.3 ± 17.8	0.44 ± 0.14	4	602
**Neuro2a cells expressing GCaMP6 plus TRPV4^{FeRIC} – Timing protocol 3 – 72 h post-transfection + holotransferrin + Fura-2**				
TRPV4^{FeRIC} - RF 1.6 μT	2.8 ± 6	0.1 ± 0.1	4	120
**Neuro2a cells expressing TRPV4^{FeRIC} loaded with Fluo-4 – Timing protocol 3 – 72 h post-transfection + holotransferrin**				
TRPV4^{FeRIC} - RF 1.6 μT	236.4 ± 40.1	0.5 ± 0.1	3	274
**Neuro2a cells expressing GCaMP6 plus TRPV4^{FeRIC} – Timing protocol 1 – 24 h post-transfection – RF at 465 kHz frequency**				
TRPV4^{FeRIC} - RF 31 μT (465 kHz)	25.6 ± 8.6	0.31 ± 0.2	5	93
**Neuro2a cells expressing GCaMP6 plus – Timing protocol 3 – 72 h post-transfection + HTF 500 μg/mL - RF at 465 kHz frequency**				
TRPV4^{FeRIC} - RF 31 μT (465 kHz)	81.4 ± 19.4	0.32 ± 0.11	5	106

Table S1. GCaMP6 and Fluo-4 data from N2a cells expressing TRPV4^{FeRIC}. Related to Figures 1 - 4. GCaMP6 and Fluo-4 data were quantified as the change in GCaMP6 fluorescence divided by baseline fluorescence (ΔF/F0). For each experimental condition, listed here is the averaged GCaMP6 or Fluo-4 area under the curve (AUC) ± SEM, the fraction of cells responsive to RF, the number of separate experiments (N), and the number of analyzed cells (n).
